# Interim results from a postmarketing surveillance study of patients with *FLT3*-mutated relapsed/refractory AML treated with the FLT3 inhibitor gilteritinib in Japan

**DOI:** 10.1093/jjco/hyac069

**Published:** 2022-05-06

**Authors:** Haruko Sugamori, Takumi Lee, Takeshi Mitomi, Chika Yamagishi

**Affiliations:** Astellas Pharma Inc., Tokyo, Japan

**Keywords:** acute myeloid leukemia, *fms*-like tyrosine kinase 3, adverse drug reaction

## Abstract

**Objective:**

Gilteritinib received approval for the treatment of *FLT3*-mutated relapsed or refractory acute myeloid leukemia in Japan in 2018. In accordance with regulatory requirements, we conducted a multicenter, observational surveillance of gilteritinib use in Japan.

**Methods:**

Patients were followed for 6 months from gilteritinib treatment initiation. The primary endpoint of the surveillance was incidence of adverse drug reactions related to each element of the safety specification defined in the Japanese Risk Management Plan. This interim analysis presents data collected from 3 December 2018 to 20 September 2020.

**Results:**

Among 204 patients with case report forms, 107 consented to data publication. Of these 107 patients, 59.8% (*n* = 64) were male and 58.9% (*n* = 63) were aged ≥65 years; most received a 120-mg/day initial (80.4%; 86/107) and maximum (74.8%; 80/107) daily dose. The discontinuation rate was 61.7% (66/107); the most common reasons for discontinuation were disease progression (18.7%), transplantation (16.8%) and adverse events (15.0%). The adverse drug reaction rate was 77.6%. The incidences of adverse drug reactions (grade ≥ 3) related to each element of the safety specification were myelosuppression, 44.9% (38.3%); liver function disorder, 24.3% (6.5%); infections, 24.3% (21.5%); prolonged QT interval, 10.3% (2.8%); hemorrhage, 9.3% (6.5%); renal dysfunction, 6.5% (0); hypersensitivity, 5.6% (1.9%); interstitial lung disease, 4.7% (3.7%); cardiac failure/pericarditis/pericardial effusion, 1.9% (0.9%); pancreatitis, 0.9% (0); posterior reversible encephalopathy syndrome, 0.9% (0.9%). The composite complete remission rate was 62.7%; the 6-month overall survival rate was 77.7%.

**Conclusion:**

Gilteritinib treatment for 6 months in Japan was associated with acceptable efficacy and no new safety concerns were observed.

## Teaser

Interim results from this Japan postmarketing surveillance study show that gilteritinib appears safe and efficacious in patients with relapsed/refractory *FLT3*-mutated AML. These data support the real-world benefit of gilteritinib.

## Introduction

Acute myeloid leukemia (AML) is a genetically heterogeneous hematologic malignancy characterized by the clonal expansion of undifferentiated myeloid precursors in the bone marrow leading to failed hematopoiesis (1). The gene encoding *fms*-like tyrosine kinase 3 (FLT3), a transmembrane cytokine receptor expressed on hematopoietic progenitor cells, is the most commonly mutated gene identified in patients with AML (2,3). The wild-type FLT3 protein functions to promote differentiation and maturation of hematopoietic progenitor cells. In contrast, activating mutations in *FLT3*, which include internal tandem duplication (ITD) mutations and tyrosine kinase domain (TKD) mutations, are associated with poor prognosis (4).

The availability of *FLT3-*targeted therapies has improved the treatment of AML. First-generation multikinase inhibitors such as midostaurin and sorafenib target FLT3 as well as other kinases. As such, these agents require administration in combination with chemotherapy to maximize their clinical benefit (5–8). More potent and FLT3-specific second-generation FLT3 inhibitors, such as gilteritinib and quizartinib, were subsequently developed and demonstrated clinical benefits in patients with *FLT3* mutation-positive (*FLT3*^mut+^) relapsed or refractory (R/R) AML (9–11). However, despite its benefit in patients with *FLT3*-ITD+ R/R AML, quizartinib is not clinically active against *FLT3*-TKD mutations, which develop as a consequence of FLT3 inhibitor therapy (12). Gilteritinib is a highly specific, oral, small molecule FLT3 inhibitor that inhibits the signaling of FLT3 derived from both *FLT3*-ITD and *FLT3*-TKD mutations, resulting in apoptosis (13).

In September 2018, gilteritinib received approval from the Japan Ministry of Health, Labour and Wealth for patients with *FLT3*^mut+^ R/R AML based on interim results from the Phase 3 ADMIRAL trial, which evaluated gilteritinib versus conventional salvage chemotherapy in this patient population (14). Subsequently, gilteritinib also received approval for this indication from the United States Food and Drug Administration (November 2018) and European Medicines Agency (October 2019) (15,16). In the ADMIRAL trial, the median age of patients in the gilteritinib arm was 62 years (range, 20–84), 39.7% of patients had primary refractory AML and 60.3% had relapsed AML. Final results from the ADMIRAL trial showed that gilteritinib-treated patients showed significantly longer overall survival (OS) than chemotherapy-treated patients (9.3 vs. 5.6 months, respectively) and achieved a 1-year OS rate of 37.1% versus 16.7% with chemotherapy (10). Rates of complete remission (CR) or CR with partial hematologic recovery were 34.0% with gilteritinib and 15.3% with salvage chemotherapy, and the median duration of gilteritinib response was 11.0 months (10). Gilteritinib treatment was also associated with a lower incidence of exposure-adjusted grade ≥ 3 adverse events (AEs) versus chemotherapy (19.34 vs. 42.44 events per patient-year, respectively) (10).

The conditions of the approval of gilteritinib in Japan based on the strategy of SAKIGAKE, the practical application of innovative medical products and devices, required an assessment of the safety of its real-world use in the postmarketing setting. The objective of this surveillance study was to assess the safety and tolerability of gilteritinib therapy in patients with R/R *FLT3*^mut+^ AML in actual clinical settings in Japan.

## Patients and methods

### Surveillance design

An all-case postmarketing drug-use surveillance of patients with R/R *FLT3*^mut+^ AML treated with commercially available gilteritinib was initiated on 3 December 2018, the start date of gilteritinib marketing in Japan. Case report forms (CRFs) were collected for all patients who had started gilteritinib therapy by 31 October 2019. This multicenter, uncontrolled, observational surveillance of gilteritinib is being conducted across 362 medical institutions in Japan. The surveillance was conducted in accordance with Good Postmarketing Study Practice in Japan to comply with data integrity standards for regulatory submission and documentation. In the international Phase 3 ADMIRAL trial, most AEs related to the safety specifications and most grade ≥ 3 AEs occurred within the first 6 months after initiation of gilteritinib therapy. Based on this finding, a 6-month observation period from the start of treatment was considered appropriate for monitoring the occurrence of AEs related to the safety specifications. As such, the 6-month observation period for each patient was initiated from the start date of treatment. For patients who died or were transferred to another hospital before the end of the 6-month surveillance, the observation period spanned from the start of treatment to the date of death or hospital transfer.

### Data collection and analysis

Patient data were collected by investigators at individual sites using CRFs for the following survey items: demographic and baseline characteristics, medical history related to AML and AML type, dosing parameters for gilteritinib, other AML therapies, AEs and adverse drug reactions (ADRs), response to treatment and transplant status. The primary endpoint was the incidence of ADRs related to each element of the safety specification defined in the Japanese Risk Management Plan, which is based on clinical trial results. The ADRs that were related to elements of the safety specification included myelosuppression, infection, hemorrhage, prolonged QT interval, cardiac failure/pericarditis/pericardial effusion, liver function disorder, renal dysfunction, gastrointestinal perforation, interstitial lung disease (ILD), hypersensitivity, pancreatitis and posterior reversible encephalopathy syndrome (PRES). Secondary endpoints were the incidence of all ADRs and serious AEs. The ADRs and AEs were categorized by preferred term and system organ class (Medical Dictionary for Regulatory Activities v23.0) and graded using National Cancer Institute Common Terminology Criteria for Adverse Events. An ADR was defined as an AE for which a causal relationship to gilteritinib could not be ruled out. Treatment response assessed using modified International Working Group criteria (17) and OS were evaluated as exploratory endpoints. The composite CR (CRc) rate was defined as the sum of patients who achieved CR, CR with incomplete hematologic recovery (CRi) and CR with incomplete platelet recovery (CRp). The cumulative continuation rate and OS rate at 1, 2, 3, 4, 5 and 6 months after initiation of gilteritinib were estimated using the Kaplan–Meier method. For patients without an event resulting in treatment discontinuation, cumulative continuation rates were calculated using the date of completion of the observation period or the date of the last visit as the end date; OS was determined using the date of completion of the observation period or the date of the last gilteritinib dose as the end date.

### Statistical analyses

Continuous variables were summarized using descriptive statistics, including mean (standard deviation), median (interquartile range) and minimum and maximum values. Discrete variables were summarized as frequencies and proportions. Data were analyzed using SAS® v9.4 (SAS Institute Inc., Cary, NC).

**Table 1:** Demographic and baseline characteristics of patients with R/R *FLT3*-mutated AML

Characteristic	***N*** = 107
**Median age, years (range)**	69 (11–85)
**Sex** Male Female	64 (59.8) 43 (40.2)
**Median body weight, kg (range)**	53.6 (25.7–95.0)
**ECOG performance status** 0–1 ≥2	82 (76.6) 25 (23.4)
**Median duration of AML, months (range)**	6.0 (0–72.0)
AML type New onset AML secondary to MDS Secondary AML	85 (79.4) 19 (17.8) 3 (2.8)
**FAB classification** M0 M1 M2 M3 M4 M5 M6 M7	5 (4.7) 20 (18.7) 29 (27.1) 0 21 (19.6) 23 (21.5) 1 (0.9) 0
**Central nervous system leukemia** No Present with symptoms Present without symptoms	95 (88.8) 2 (1.9) 3 (2.8)
***FLT3* mutation type, *n* (%)** *FLT3*-ITD only *FLT3*-TKD only *FLT3*-ITD and *FLT3*-TKD Other	82 (76.6) 8 (7.5) 5 (4.7) 12 (11.2)
**Response to AML treatment before initiation of gilteritinib** Relapsed Refractory	46 (43.0) 61 (57.0)
**AML treatment immediately before initiation of gilteritinib** Remission induction Consolidation Salvage Other	68 (63.6) 6 (5.6) 24 (22.4) 9 (8.4)
**Prior HSCT** Yes No	21 (19.6) 86 (80.4)
**Presence of comorbidities** Yes No	84 (78.5) 23 (21.5)

Data are reported as *n* (%) unless otherwise noted.

AML, acute myeloid leukemia; ECOG, Eastern Cooperative Oncology Group; FAB, French–American–British; HSCT, hematopoietic stem cell transplantation; ITD, internal tandem duplication; MDS, myelodysplastic syndrome; R/R, relapsed or refractory; TKD, tyrosine kinase domain.

## Results

### Patient disposition and baseline characteristics

A total of 328 patients have been enrolled between the date of initiation of surveillance on 3 December 2018 and 20 September 2020 (end of surveillance for the fourth safety update report). Of the 204 patients with complete CRFs, 107 patients across 81 sites provided consent to publish survey results and were included in the safety and efficacy analysis sets.

Of these 107 patients, most were male (59.8%; *n* = 64), aged ≥65 years (58.9%; *n* = 63) and had an Eastern Cooperative Oncology Group performance status of 0–1 (76.6%; *n* = 82) (Table 1). Overall, 57% (*n* = 61/107) of patients had refractory AML and 78.5% (*n* = 84/107) had comorbidities, with hypertension (29.9%; *n* = 32/107), diabetes mellitus (13.1%; *n* = 14/107), dyslipidemia (12.1%; *n* = 13/107) and pneumonia (10.3%; *n* = 11/107) being the most common. Overall, 76.6% (*n* = 82/107) had *FLT3*-ITD mutations only, 7.5% (*n* = 8/107) had *FLT3*-TKD mutations only, 4.7% (*n* = 5/107) had both *FLT3*-ITD and *FLT3*-TKD mutations and 11.2% (*n* = 12/107) had other *FLT3* mutations. Although gilteritinib is not indicated for use in pediatric patients, six patients aged 11–14 years received gilteritinib off label. All six pediatric patients had *FLT3*-ITD mutations without *FLT3*-TKD point mutations, and three had relapsed after prior hematopoietic stem cell transplantation (HSCT). Of the 107 patients evaluated, 19 (male, *n* = 15; female, *n* = 4) had AML secondary to myelodysplastic syndrome (MDS); all but one was *FLT3*-ITD+, 14 had refractory disease and five had relapsed AML. Thirteen of these 19 patients had previously received high-intensity chemotherapy.

At the time of treatment initiation with gilteritinib, five patients were diagnosed with central nervous system (CNS) leukemia; two were symptomatic. One case of symptomatic CNS leukemia was observed in a 69-year-old female patient with *FLT3*-ITD+ AML who displayed symptoms of peripheral neuropathy, insomnia, chronic gastritis, chronic enteritis and constipation. The second case of symptomatic CNS leukemia was observed in a heavily pretreated 70-year-old male patient with *FLT3*-ITD+ AML secondary to MDS. The patient had symptomatic epilepsy with CNS infiltration.

### Treatment with gilteritinib

During the surveillance period, most patients received 120-mg gilteritinib as the initial daily dose (80.4%; *n* = 86/107) as well as the maximum daily dose (74.8%; *n* = 80/107) (Table 2). Approximately 52% (*n* = 56/107) of patients required dose modifications, most frequently within the first 2 months of treatment. Discontinuation of gilteritinib was observed in 61.7% (*n* = 66/107) of patients; the most common reasons for discontinuation were disease progression (18.7%; *n* = 20), transplantation (16.8%; *n* = 18) and AEs (15.0%; *n* = 16). Initial doses for two pediatric patients weighing <30 kg were 70 mg in an 11-year-old male patient and 40 mg in a 12-year-old female patient; the dose of gilteritinib was subsequently increased to 120 mg in the 12-year-old patient following disease progression.

**Table 2:** Gilteritinib exposure in patients with R/R *FLT3*-mutated AML

Parameter	***N*** = 107
**Initial daily dose** 40 mg 80 mg 120 mg 160 mg 200 mg Other	10 (9.3) 10 (9.3) 86 (80.4) 0 0 1 (0.9)
**Maximum daily dose** 40 mg 80 mg 120 mg 160 mg 200 mg Other	3 (2.8) 11 (10.3) 80 (74.8) 2 (1.9) 10 (9.3) 1 (0.9)
**Median dosing duration, days (range)**	105 (3–181)
**Drug withdrawal** Yes No	42 (39.3) 65 (60.7)
**Dose modification** Yes No	56 (52.3) 51 (47.7)
**Time from initial dose to dose modification** <1 month ≥1 month–<2 months ≥2 months–<3 months ≥3 months–<4 months ≥4 months–<5 months ≥5 months–<6 months ≥6 months	21 (19.6) 16 (15.0) 4 (3.7) 5 (4.7) 5 (4.7) 5 (4.7) 0
**Discontinuation** Yes No	66 (61.7) 41 (38.3)
**Reasons for discontinuation** Disease progression Transplantation Adverse event Other Hospital transfer Death Patient request Relapse	20 (18.7) 18 (16.8) 16 (15.0) 4 (3.7) 4 (3.7) 3 (2.8) 3 (2.8) 1 (0.9)

Data are reported as *n* (%) unless otherwise noted.

AML, acute myeloid leukemia; R/R, relapsed or refractory.

**Figure 1:**
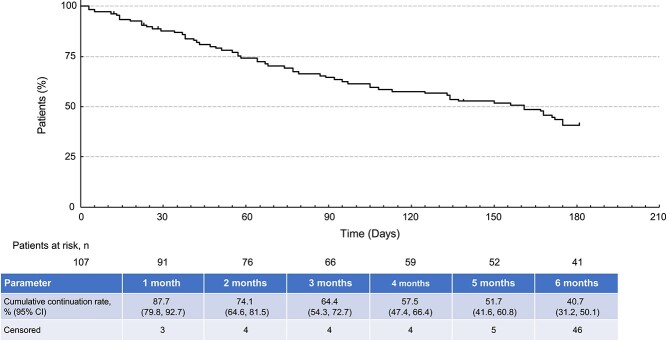
Cumulative continuation rate of gilteritinib during the surveillance period. Abbreviation: CI, confidence interval.

The cumulative continuation rate of gilteritinib over the 6-month surveillance period is shown in Fig. 1**.** The cumulative continuation rate declined to 40.7% over the 6-month period.

### Other treatments for AML

Of the 107 patients receiving gilteritinib, 12 (11.2%) also received other AML therapies, which included antimetabolites (*n* = 10; 9.3%), antineoplastic antibiotics (*n* = 4; 3.7%), plant alkaloids (*n* = 3; 2.8%), other antitumor drugs [mitoxantrone, *n* = 1 (0.9%); azacitidine, *n* = 1 (0.9%)] and alkylating agents (*n* = 1; 0.9%). Six of these 12 patients received concomitant treatment for AML with other agents: five received concomitant treatment with antimetabolites (cytarabine, methotrexate or hydroxycarbamide) and one received concomitant treatment with azacitidine.

### Safety outcomes

Overall, 77.6% (*n* = 83/107) of patients experienced ADRs during the surveillance period. The incidence of ADRs related to each element of the safety specification were as follows: myelosuppression (44.9%; *n* = 48/107), liver function disorder (24.3%; *n* = 26/107), infections (24.3%; *n* = 26/107), prolonged QT interval (10.3%; *n* = 11/107), hemorrhage (9.3%; *n* = 10/107), renal dysfunction (6.5%; *n* = 7/107), hypersensitivity (5.6%; *n* = 6/107), ILD (4.7%; *n* = 5/107), cardiac failure/pericarditis/pericardial effusion (1.9%; *n* = 2/107), pancreatitis (0.9%; *n* = 1/107) and PRES (0.9%; *n* = 1/107) (Table 3). There was no incidence of gastrointestinal perforation, which was observed as an important identified risk based on results from clinical studies of gilteritinib (10,18,19) and safety monitoring activities. At the time of this interim analysis, most ADRs related to myelosuppression (*n* = 32/48), liver function disorder (*n* = 23/26), infections (*n* = 21/26) and prolonged QT interval (*n* = 10/11) had either resolved or were in the process of resolving in most patients. The most common fatal ADRs were related to infections (2.8%; *n* = 3/107) and hemorrhage (2.8%; *n* = 3/107).

**Table 3:** Incidence of ADRs for each safety specification

Safety Specification	Any^a^^,^^b^	Grade ≥ 3^a^^,^^b^	Resolved/ Resolving^c^	Not Resolved^c^	Death^c^	Median Time to Onset, Days (Range)	Median Time to Resolution, Days (Range)
Myelosuppression^c^	48 (44.9)	41 (38.3)	32 (66.7)	14 (29.2)	1 (2.1)	9 (1–113)	21.5 (3–211)
Infections	26 (24.3)	23 (21.5)	21 (80.8)	2 (7.7)	3 (11.5)	36 (4–128)	18 (9–57)
Liver function disorder	26 (24.3)	7 (6.5)	23 (88.5)	3 (11.5)	0	18.5 (3–166)	34 (6–255)
Prolonged QT interval	11 (10.3)	3 (2.8)	10 (90.9)	1 (9.1)	0	16 (4–122)	5 (2–129)
Hemorrhage	10 (9.3)	7 (6.5)	6 (60.0)	1 (10.0)	3 (30.0)	62.5 (3–109)	42 (22–157)
Renal dysfunction	7 (6.5)	0	6 (85.7)	1 (14.3)	0	27 (4–112)	82.5 (7–239)
Hypersensitivity	6 (5.6)	2 (1.9)	6 (100)	0	0	33 (1–60)	12.5 (8–41)
Interstitial lung disease	5 (4.7)	4 (3.7)	4 (80.0)	0	1 (20.0)	58 (20–141)	18.5 (10–61)
Cardiac failure/pericarditis/pericardial effusion	2 (1.9)	1 (0.9)	2 (100)	0	0	42.5 (25–59)	50.5 (43–58)
PRES	1 (0.9)	1 (0.9)	1 (100)	0	0	25 (25–25)	3 (3–3)
Pancreatitis	1 (0.9)	0	0	1 (100)	0	70 (70–70)	NA
Gastrointestinal perforation	0	0	0	0	0	NA	NA

Values are reported as *n* (%) unless otherwise noted.

^a^Percentage was calculated using a denominator of 107.

^**b**^If the same ADR was observed multiple times after gilteritinib administration in the same patient, the earliest occurring ADR is reported here.

^c^The outcome was unknown for one patient who experienced myelosuppression.

ADR, adverse drug reaction; NA, not applicable; PRES, posterior reversible encephalopathy syndrome.

Supplementary Table S1 shows a complete list of each safety specification classified by the Medical Dictionary for Regulatory Activities (MedDRA) preferred term and the time-to-onset and time to resolution of ADRs and action taken with respect to gilteritinib therapy. Specific ADRs leading to dose reductions in more than one patient were decreased platelet count (*n* = 3), bone marrow failure (*n* = 3), prolonged QT interval (*n* = 2) and abnormal liver function (*n* = 2) (Supplementary Table S1). Specific ADRs leading to drug withdrawal in more than one patient were decreased platelet count (*n* = 6), prolonged QT interval (*n* = 6), abnormal liver function (*n* = 4), decreased white blood cell count (*n* = 4), pneumonia (*n* = 3), sepsis (*n* = 2), hemorrhage (*n* = 2), ILD (*n* = 2) and renal disorder (*n* = 2); ADRs leading to discontinuation of gilteritinib in more than one patient were febrile neutropenia (*n* = 2), neutropenia (*n* = 2), cellulitis (*n* = 2) and sepsis (*n* = 2) (Supplementary Table S1).

Among the 19 patients with AML secondary to MDS, 10 experienced grade ≥ 3 myelosuppression; two cases of myelosuppression were suspected to be related to gilteritinib. In four of the 10 patients, gilteritinib dose was either reduced or treatment was discontinued.

Grade 3 ILD was observed in two patients. One patient was a 35-year-old man who had relapsed after transplantation with chronic graft-versus-host disease (GVHD). Twenty days after initiation of 120-mg gilteritinib, the patient developed Grade 3 ILD. Gilteritinib was withdrawn and the patient was treated with a steroid pulse and recovered over a 10-day period. The patient subsequently underwent a second transplantation. The cause of ILD was thought to be related to both gilteritinib and GVHD. The other patient who developed Grade 3 ILD was a 71-year-old man who had a history of lung cancer and mantle cell lymphoma. He developed Grade 3 ILD 58 days from initiation of 120-mg gilteritinib. Although remaining on gilteritinib therapy, the patient received prednisone and recovered over a 13-day period. The cause of ILD in this patient was thought to be related to gilteritinib and vancomycin.

A 79-year-old male patient with refractory AML secondary to MDS, who had a history of gastric cancer and prostate cancer, developed Grade 2 pancreatitis during treatment with gilteritinib. The patient had initiated gilteritinib therapy at a dose of 120 mg and the dose was subsequently increased to 200 mg due to AML progression. Seventy days after initiation of gilteritinib treatment, the patient developed Grade 2 pancreatitis that was not considered to be serious. The patient continued gilteritinib therapy. A 76-year-old female patient developed a rash that was diagnosed as Grade 3 Sweet syndrome 41 days after initiation of 120-mg gilteritinib. Treatment with gilteritinib was stopped and the patient was treated with prednisone. The patient resumed gilteritinib at an initial dose of 80 mg that was subsequently increased to 120 mg. The rash resolved and did not recur after resumption of gilteritinib.

**Figure 2:**
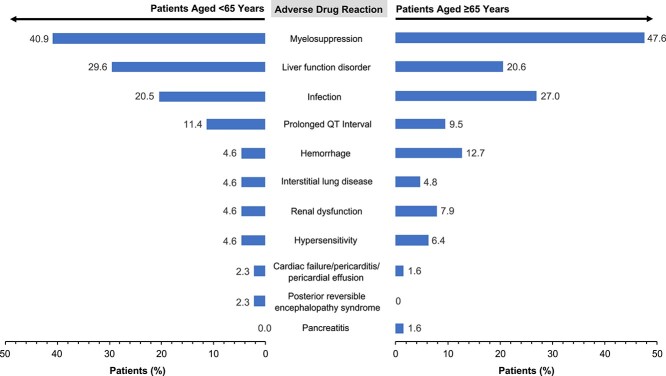
Adverse drug reactions occurring in ≥2% of patients <65 years and ≥65 years.

The incidences of all ADRs as secondary endpoints are shown in Supplementary Table S2. There was no marked difference in the incidence of ADRs between patients aged <65 years and those aged ≥65 years (Fig. 2). Serious AEs occurred in 55.1% of patients (*n* = 59/107); the most frequent were bone marrow failure (12.1%), febrile neutropenia (10.3%), decreased platelet count (7.5%) and pneumonia (5.6%) (Supplementary Table S3).

### Response outcomes

Among patients who were assessed for treatment response (*n* = 67), 34.3% achieved CR and 62.7% achieved CRc (Table 4); the median time to reach CR or CRc was similar (1.8 and 1.6 months, respectively). Four of the six pediatric patients responded to treatment with gilteritinib, with three achieving CR and one achieving CRp. A total of 17 adult patients received starting gilteritinib doses of 40 mg (*n* = 9) or 80 mg (*n* = 8); the dose was subsequently increased to 120 mg in four of these patients. Response outcomes were reported for seven of the 17 patients and included CR (*n* = 1), CRi (*n* = 3), partial remission (PR; *n* = 2) and no response (NR; *n* = 1).

**Table 4:** Response outcomes in patients receiving gilteritinib during the surveillance period

Response Parameter	*N* = 67^a^
Best response, *n* (%) CR	23 (34.3)
CRi	16 (23.9)
CRp	3 (4.5)
PR	14 (20.9)
NR	8 (11.9)
NA	3 (4.5)
***CRc*** ^***b***^	***42 (62.7)***
***ORR*** ^***c***^	***56 (83.6)***
Median time to CR, months (IQR)	1.8 (0.9–3.6)
Median time to CRc, months (IQR)	1.6 (0.9–3.0)
Median time to response, months (IQR)	1.3 (0.9–2.4)

Bold italicized font indicates aggregate response rates.

^a^Patients in the efficacy analysis set who had an assessment of treatment response (CR, CRi, CRp, PR, NR or NA).

^b^Defined as the sum of patients who achieved CR, CRi or CRp.

^c^Defined as the sum of patients who achieved CRc and PR.

CR, complete remission; CRc, composite complete remission; CRi, complete remission with incomplete hematologic recovery; CRp, complete remission with incomplete platelet recovery; IQR, interquartile range; NA, not assessable; NR, no response; ORR, overall response rate; PR, partial remission.

Response outcomes were assessed in seven of the 12 patients who received both gilteritinib and chemotherapy during the gilteritinib treatment period. Three of these seven patients had received chemotherapy (azacitidine, cytarabine or hydroxycarbamide) concomitantly with gilteritinib, with responses of PR (*n* = 1) or NR (*n* = 2). In the remaining four patients, chemotherapy [daunorubicin (*n* = 1), aclarubicin plus cytarabine once and an *A-triple V* (cytarabine, etoposide, vincristine and vindesine) regimen twice (*n* = 1), cytarabine plus idarubicin and mitoxantrone (*n* = 1) or cytarabine (*n* = 1)] was initiated after treatment with gilteritinib was temporarily stopped; gilteritinib was restarted after chemotherapy was stopped. Responses in these four patients were CR (*n* = 2), PR (*n* = 1) and NR (*n* = 1).

**Figure 3:**
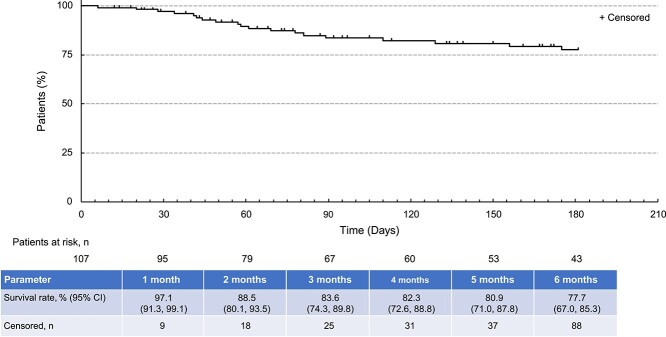
Overall survival in patients receiving gilteritinib during the surveillance period. Abbreviation: CI, confidence interval.

After an initial decline during the first 3 months of surveillance, OS rates remained relatively stable until the end of the surveillance period (Fig. 3).

### Patients who underwent transplantation

A total of 14 patients underwent HSCT during the surveillance period (Supplementary Table S4). Six of the 14 patients who underwent HSCT had received prior HSCT and two had GVHD at the start of gilteritinib therapy. Sources of hematopoietic stem cells were peripheral blood (42.9%), umbilical cord blood (28.6%) and bone marrow (28.6%). Engraftment was observed in all but one patient (Supplementary Table S4). Of the 11 patients who had been assessed for response before HSCT, five achieved CR, five achieved CRi and one achieved CRp.

Six patients resumed gilteritinib after HSCT (Table 5); the median time from HSCT to the restart of gilteritinib therapy was 37 days (range, 24–102 days). Three of the six patients restarted gilteritinib at the 40-mg dose, one resumed gilteritinib at the 80-mg dose and the remaining two patients resumed gilteritinib at the 120-mg dose (Table 5). In two patients who each resumed gilteritinib at 40 and 80 mg, the dose was increased to 120 mg over a 2-week period. AEs occurred in three of the six patients who resumed gilteritinib after HSCT. Two of these patients had restarted gilteritinib at doses of 40 mg and 120 mg, respectively, and both patients developed liver dysfunction that was suspected to be related to gilteritinib (low possibility of causal relationship; *n* = 1) or liver GVHD (relationship to gilteritinib was not evaluable; *n* = 1). The third patient developed sepsis (unrelated to gilteritinib) before restarting gilteritinib therapy.

**Table 5:** Gilteritinib dosing parameters before and after transplantation

Dosing Before HSCT	*N* = 14
Median duration of dosing before HSCT, days (range)	93 (24–176)
**Dosing After HSCT**	***N* = 6**
Daily dose immediately before HSCT, *n* (%) 40 mg 80 mg 120 mg	0 0 6 (100)
Daily dose immediately after HSCT, *n* (%) 40 mg 80 mg 120 mg	3 (50) 1 (16.7) 2 (33.3)
Median time to restart of gilteritinib after HSCT, days (range)	37 (24–102)

HSCT, hematopoietic stem cell transplantation.

## Discussion

A limited number of patients with R/R *FLT3*^mut+^ AML participated in gilteritinib clinical trials in Japan. Thus, the regulatory authority required a postmarketing use-results surveillance covering all patients treated with gilteritinib to obtain patient characteristics, gilteritinib safety and efficacy and measures taken to ensure proper use of gilteritinib. The results of this interim analysis show that real-world use of gilteritinib in Japan was not associated with any new safety concerns and was effective in a variety of patients with R/R *FLT3*^mut+^ AML. At enrollment, >50% of patients had refractory AML and >75% had comorbidities, indicating that this study included patients with multiple comorbidities and those who were ineligible for participation in a clinical trial. The 120-mg/day dose, which was identified in dose-escalation studies as the preferred starting dose (18,19), was used by the majority of patients in this study as the initial dose (80.4%; 86/107) and the maximum dose (74.8%; 80/107). Although gilteritinib is not indicated for pediatric patients with R/R *FLT3*^mut+^ AML, it was administered off-label in six pediatric patients at ages ranging from 11 to 14 years.

Overall, the safety profile of gilteritinib observed during the surveillance period did not show any new clinically significant safety signals. Due to the impact of persistent disease, cytopenias and myelosuppression are commonly observed in patients with AML. In general, most ADRs related to myelosuppression, liver function disorder, infections and prolonged QT interval were manageable and had either resolved or were in the process of resolving in a majority of patients at the time of this interim analysis. The proportion of patients who discontinued gilteritinib due to an AE was low (15.0%). Overall, common ADRs were reported less frequently in this survey than in the gilteritinib clinical trials (10,18,19). It should be noted that mild or moderate liver dysfunction does not have a significant effect on the pharmacokinetic parameters of gilteritinib (16). In pharmacokinetic analyses, the ratio of the maximum plasma concentration (C_max_) of unbound gilteritinib in patients with moderate hepatic impairment (Child-Pugh B) to the C_max_ of unbound gilteritinib in healthy volunteers was 117.72% [90% confidence interval (CI): 89.90, 154.15]; the corresponding ratio for area under the plasma concentration–time curve from time 0 to infinity was 88.48% (90% CI: 65.97, 118.69) (20). Elevated AST or ALT was found to be mildly correlated with gilteritinib plasma concentration (21).

The rate of CRc in this surveillance (62.7%) was slightly higher than that observed in the Phase 3 clinical trial of gilteritinib (54.3%) (10). The change in OS rate over the 6-month surveillance period was $\sim$20%, with $\sim$78% of patients remaining alive at the end of surveillance. Of the 14 patients who underwent HSCT, all but one had engraftment, and six resumed gilteritinib treatment posttransplantation. All patients who were assessed for response before undergoing HSCT (*n* = 11) had achieved CRc before transplantation.

These findings are limited in that they are based on the interim analysis prespecified in the study protocol. Thus, the results may change with the collection of additional data. In addition, only data from the 107 patients who provided informed consent were tabulated and analyzed for this report; thus, they may not be representative of the entire population surveyed (*N* = 328) or of the 204 patients with CRFs. Because only 67 of the 107 patients in this analysis had an assessment of treatment response, a selection bias toward patients with a documented response cannot be ruled out. Furthermore, the reported efficacy data included the effectiveness of gilteritinib in patients who received low, off-label doses and the effectiveness of other drugs in addition to gilteritinib alone. With regard to remission rate, the period from initiation of treatment to the time of response assessment varied among patients. Finally, survival was calculated with the date of discontinuation of gilteritinib as the end date, even in surviving patients.

In conclusion, interim results from this postmarketing surveillance study of gilteritinib suggest that gilteritinib has acceptable safety and efficacy in patients with R/R *FLT3*^mut+^ AML in actual use settings in Japan. These findings confirm the clinical benefit provided by gilteritinib in a real-world R/R *FLT3*^mut+^ AML patient population.

## Supplementary Material

Japan_Postmarketing_Ms_SUPPLEMENTARY_MATERIALv2_hyac069

## Data Sharing Statement

Researchers may request access to anonymized participant level data, trial level data and protocols from Astellas sponsored clinical trials at www.clinicalstudydatarequest.com.

For the Astellas criteria on data sharing see: https://clinicalstudydatarequest.com/Study-Sponsors/Study-Sponsors-Astellas.aspx.
