# Supplementary material for: Interim results from a postmarketing surveillance study of patients with FLT3-mutated relapsed/refractory AML treated with the FLT3 inhibitor gilteritinib in Japan
Source: Jpn J Clin Oncol. 2022 May 6;52(7):766–73. doi: 10.1093/jjco/hyac069 (PMC9264337; doi:10.1093/jjco/hyac069)
Supplement: Japan_Postmarketing_Ms_SUPPLEMENTARY_MATERIALv2_hyac069 [file japan_postmarketing_ms_supplementary_materialv2_hyac069.docx]

**SUPPLEMENTARY MATERIAL**

**Table S1. Onset of ADRs in each safety specification and action taken with gilteritinib**

| **ADRs^*^** | **Time to Onset** | | **Time to Resolution** | | **Gilteritinib Administration** | | | | |
| --- | --- | --- | --- | --- | --- | --- | --- | --- | --- |
|  | **Number of Patients** | **Median Time, Days (range)** | **Number of Patients** | **Median Time, Days (range)** | **Continued^†,‡^,**  ***n* (%)** | **Dose Reduced^†,‡^,**  ***n* (%)** | **Drug Withdrawal^†,‡^,**  ***n* (%)** | **Discontinued^†,‡^,**  ***n* (%)** | **Discontinued Before AE Onset^†,‡^, *n* (%)** |
| **Myelosuppression (*n*=48)** | | | | | | | | | |
| **Any ADR** | 48 | 9 (1-113) | 32 | 21.5 (3-211) | **25 (52.1)** | **5 (10.4)** | **14 (29.2)** | **4 (8.3)** | **0** |
| Bone marrow failure  Platelet count decreased  Febrile neutropenia  Neutrophil count decreased  Anemia  Pancytopenia  Neutropenia  White blood cell count decreased  Thrombocytopenia | 15  17  10  7  4  3  5  7  2 | 8 (1-113)  21 (3-61)  10 (1-113)  19 (9-36)  3 (1-103)  1 (1-3)  8 (1-97)  50 (6-97)  12.5 (5-20) | 10  12  9  5  2  0  4  5  1 | 21 (8-100)  29.5 (8-211)  28 (4-109)  25 (3-84)  216 (32-400)  -  20 (9-83)  14 (9-64)  23 (23-23) | 9 (60.0)  8 (47.1)  7 (70.0)  6 (85.7)  3 (75.0)  2 (66.7)  2 (40.0)  2 (28.6)  0 | 3 (20.0)  3 (17.6)  0  0  0  1 (33.3)  0  0  0 | 2 (13.3)  6 (35.3)  1 (10.0)  1 (14.3)  0  0  1 (20.0)  4 (57.1)  1 (50.0) | 1 (6.7)  0  2 (20.0)  0  1 (25.0)  0  2 (40.0)  1 (14.3)  1 (50.0) | 0  0  0  0  0  0  0  0  0 |
| **Infections (*n*=26)** | | | | | | | | | |
| **Any ADR** | **26** | **36 (4-128)** | **21** | **18 (9-57)** | **13 (50.0)** | **0** | **6 (23.1)** | **7 (26.9)** | **0** |
| Infections  Pneumonia  Bacteremia  Bronchitis  Bronchopulmonary aspergillosis  Cellulitis  Cystitis  Cytomegalovirus infection  Herpes zoster infection  Streptococcal sepsis  Staphylococcal infection  Infective spondylitis  Mycotic endophthalmitis  Device-related infection  Pneumocystis jiroveci pneumonia  Campylobacter colitis  Fungal pneumonia  Sepsis  Septic shock | 3  6  1  1  1  4  1  1  1  1  1  1  1  1  1  1  1  4  1 | 99 (34-128)  25.5 (5-77)  161 (161-161)  31 (31-31)  58 (58-58)  35.5 (15-49)  123 (123-123)  33 (33-33)  116 (116-116)  10 (10-10)  7 (7-7)  143 (143-143)  37 (37-37)  35 (35-35)  49 (49-49)  49 (49-49)  60 (60-60)  44.5 (4-85)  57 (57-57) | 3  5  1  1  0  3  1  1  1  0  1  1  1  1  1  1  0  3  1 | 37 (9-57)  42 (18-50)  67 (67-67)  12 (12-12)  -  11 (9-13)  14 (14-14)  14 (14-14)  6 (6-6)  -  36 (36-36)  39 (39-39)  19 (19-19)  37 (37-37)  23 (23-23)  9 (9-9)  -  15 (10-17)  16 (16-16) | 3 (100)  2 (33.3)  1 (100)  1 (100)  1 (100)  1 (25.0)  1 (100)  1 (100)  1 (100)  1 (100)  1 (100)  1 (100)  1 (100)  1 (100)  1 (100)  1 (100)  0  0  0 | 0  0  0  0  0  0  0  0  0  0  0  0  0  0  0  0  0  0  0 | 0  3 (50.0)  0  0  0  1 (25.0)  0  0  0  0  0  0  0  0  0  0  0  2 (50.0)  0 | 0  1 (16.7)  0  0  0  2 (50.0)  0  0  0  0  0  0  0  0  0  0  1 (100)  2 (50.0)  1 (100) | 0  0  0  0  0  0  0  0  0  0  0  0  0  0  0  0  0  0  0 |
| **Liver function disorder (*n*=26)** | | | | | | | | | |
| **Any ADR** | **26** | **18.5 (3-166)** | **23** | **34 (6-225)** | **17 (65.4)** | **2 (7.7)** | **6 (23.1)** | **1 (3.8)** | **0** |
| Abnormal liver function  AST increased  Liver enzyme increased  ALT increased  ALT abnormal  AST abnormal  Gamma-glutamyl transferase increased | 17  4  4  3  1  1  1 | 27 (3-166)  9.5 (4-46)  13 (8-101)  21 (4-25)  5 (5-5)  5 (5-5)  31 (31-31) | 15  3  4  3  1  1  1 | 29 (6-225)  75 (8-87)  79.5 (36-239)  77 (8-78)  25 (25-25)  25 (25-25)  29 (29-29) | 10 (58.8)  4 (100)  3 (75.0)  2 (66.7)  1 (100)  1 (100)  1 (100) | 2 (11.8)  0  0  0  0  0  0 | 4 (23.5)  0  1 (25.0)  1 (33.3)  0  0  0 | 1 (5.9)  0  0  0  0  0  0 | 0  0  0  0  0  0  0 |
| **Prolonged QT interval (*n*=11)** | | | | | | | | | |
| **Any ADR** | **11** | **16 (4-122)** | **10** | **5 (2-129)** | **2 (18.2)** | **2 (18.2)** | **6 (54.5)** | **1 (9.1)** | **0** |
| Electrocardiogram QT prolonged | 11 | 16 (4-122) | 10 | 5 (2-129) | 2 (18.2) | 2 (18.2) | 6 (54.5) | 1 (9.1) | 0 |
| **Hemorrhage (*n*=10)** | | | | | | | | | |
| **Any ADR** | **10** | **62.5 (3-109)** | **6** | **42 (22-157)** | **4 (40.0)** | **1 (10.0)** | **2 (20.0)** | **1 (10.0)** | **2 (20.0)** |
| Cerebral hemorrhage  Gastrointestinal hemorrhage  Hematuria  Pulmonary alveolar hemorrhage  Subcutaneous hematoma  Lower gastrointestinal hemorrhage  Hemorrhage  Mucosal hemorrhage | 2  2  1  1  1  1  1  1 | 24.5 (5-44)  92 (77-107)  3 (3-3)  109 (109-109)  80 (80-80)  48 (48-48)  25 (25-25)  85 (85-85) | 0  2  1  0  1  0  1  1 | -  39.5 (36-43)  41 (41-41)  -  22 (22-22)  -  157 (157-157)  56 (56-56) | 0  0  1 (100)  0  1 (100)  1 (100)  0  1 (100) | 0  0  0  0  0  0  1 (100)  0 | 0  2 (100)  0  0  0  0  0  0 | 1 (50.0)  0  0  0  0  0  0  0 | 1 (50.0)  0  0  1 (100)  0  0  0  0 |
| **Renal dysfunction (*n*=7)** | | | | | | | | | |
| **Any ADR** | **7** | **27 (4-112)** | **6** | **82.5 (7-239)** | **4 (57.1)** | **1 (14.3)** | **2 (28.6)** | **0** | **0** |
| Blood creatinine increased  Renal impairment | 2  5 | 52.5 (4-101)  27 (4-112) | 2  4 | 127 (15-239)  82.5 (7-124) | 2 (100)  2 (40.0) | 0  1 (20.0) | 0  2 (40.0) | 0  0 | 0  0 |
| **Hypersensitivity (*n*=6)** | | | | | | | | | |
| **Any ADR** | **6** | **33 (1-60)** | **6** | **12.5 (8-41)** | **4 (66.7)** | **0** | **1 (16.7)** | **1 (16.7)** | **0** |
| Erythema multiforme  Face edema  Rash  Rash maculopapular | 1  1  3  1 | 60 (60-60)  8 (8-8)  43 (1-57)  23 (23-23) | 1  1  3  1 | 15 (15-15)  8 (8-8)  21 (10-41)  10 (10-10) | 0  1 (100)  3 (100)  0 | 0  0  0  0 | 1 (100)  0  0  0 | 0  0  0  1 (100) | 0  0  0  0 |
| **Interstitial lung disease (*n*=5)** | | | | | | | | | |
| **Any ADR** | **5** | **58 (20-141)** | **4** | **18.5 (10-61)** | **2 (40.0)** | **0** | **2 (40.0)** | **0** | **1 (20.0)** |
| Interstitial lung disease  Pulmonary alveolar hemorrhage  Organizing pneumonia  Lung opacity | 2^§^  1  1  1 | 39 (20-58)  109 (109-109)  29 (29-29)  141 (141-141) | 2^§^  0  1  1 | 11.5 (10-13)  -  61 (61-61)  24 (24-24) | 1^§^ (50.0)  0  0  1 (100) | 0  0  0  0 | 1^§^ (50.0)  0  1 (100)  0 | 0  0  0  0 | 0  0  0  1 (100) |
| **Cardiac failure/pericarditis/pericardial effusion (*n*=2)** | | | | | | | | | |
| **Any ADR** | **2** | **42.5 (26-59)** | **2** | **50.5 (43-58)** | **1 (50.0)** | **0** | **1 (50.0)** | **0** | **0** |
| Cardiac failure | 2 | 42.5 (26-59) | 2 | 50.5 (43-58) | 1 (50.0) | 0 | 1 (50.0) | 0 | 0 |
| **Posterior reversible encephalopathy syndrome (*n*=1)** | | | | | | | | | |
| **Any ADR** | **1** | **25 (25-25)** | **1** | **3 (3-3)** | **0** | **1 (100)** | **0** | **0** | **0** |
| Posterior reversible encephalopathy syndrome | 1 | 25 (25-25) | 1 | 3 (3-3) | 0 | 1 (100) | 0 | 0 | 0 |
| **Pancreatitis (n=1)** | | | | | | | | | |
| **Any ADR** | **1** | **70 (70-70)** | **0** | **-** | **1 (100)** | **0** | **0** | **0** | **0** |
| Pancreatitis | 1 | 70 (70-70) | 0 | - | 1 (100) | 0 | 0 | 0 | 0 |

^*^Some patients had more than one adverse drug reaction.

^†^Percentages for bold values corresponding to “Any ADR” reported using the total number of patients for each safety specification as the denominator.

^‡^The percentages for specific ADRs are reported using the total number of patients with that ADR as the denominator.

^§^Patients with interstitial lung disease are described in the results section.

ADR, adverse drug reaction; AE, adverse event; ALT, alanine aminotransferase; AST, aspartate aminotransferase.

**Table S2. Incidence of ADRs by SOC and preferred term**

| **ADRs, *n* (%)** | ***N*=107** |
| --- | --- |
| Any ADR | 83 (77.6) |
| **Investigations** | **46 (43.0)** |
| Platelet count decreased  Electrocardiogram QT prolonged  Neutrophil count decreased  White blood cell count decreased  Blood creatinine phosphokinase increased  Blood lactate dehydrogenase increased  Hepatic enzyme increased  AST increased  ALT increased  Amylase increased  Blood creatinine increased  Blood alkaline phosphatase increased  ALT abnormal  AST abnormal  Gamma-glutamyl transferase increased  Hemoglobin decreased  Lymphocyte count increased  Aspergillus test positive  C-reactive protein increased | 17 (15.9)  11 (10.3)  7 (6.5)  7 (6.5)  5 (4.7)  5 (4.7)  4 (3.7)  4 (3.7)  3 (2.8)  3 (2.8)  2 (1.9)  2 (1.9)  1 (0.9)  1 (0.9)  1 (0.9)  1 (0.9)  1 (0.9)  1 (0.9)  1 (0.9) |
| **Blood and lymphatic system disorders** | **29 (27.1)** |
| Bone marrow failure  Febrile neutropenia  Neutropenia  Pancytopenia  Thrombocytopenia  Eosinophilia | 15 (14.0)  10 (9.3)  5 (4.7)  3 (2.8)  2 (1.9)  1 (0.9) |
| **Hepatobiliary disorders** | **28 (26.2)** |
| Liver function abnormal  Liver disorder  Cholangitis  Hepatic steatosis | 17 (15.9)  10 (9.3)  1 (0.9)  1 (0.9) |
| **Infections and infestations** | **26 (24.3)** |
| Pneumonia  Sepsis  Cellulitis  Infections  Bacteremia  Bronchitis  Bronchopulmonary aspergillosis  Cystitis  Cytomegalovirus infection  Herpes zoster infection  Septic shock  Pneumonia fungal  Infective spondylitis  Mycotic endophthalmitis  Device related infection  Pneumocystis jirovecii pneumonia  Campylobacter colitis | 6 (5.6)  4 (3.7)  4 (3.7)  3 (2.8)  1 (0.9)  1 (0.9)  1 (0.9)  1 (0.9)  1 (0.9)  1 (0.9)  1 (0.9)  1 (0.9)  1 (0.9)  1 (0.9)  1 (0.9)  1 (0.9)  1 (0.9) |
| **Gastrointestinal disorders** | **9 (8.4)** |
| Gastrointestinal hemorrhage  Nausea  Lower gastrointestinal hemorrhage  Abdominal pain  Abdominal pain lower  Constipation  Diarrhea  Enterocolitis  Pancreatitis | 2 (1.9)  2 (1.9)  1 (0.9)  1 (0.9)  1 (0.9)  1 (0.9)  1 (0.9)  1 (0.9)  1 (0.9) |
| **Respiratory, thoracic, and mediastinal disorders** | **9 (8.4)** |
| Interstitial lung disease  Cough  Dyspnea  Pneumonia aspiration  Pulmonary alveolar hemorrhage  Upper respiratory tract inflammation  Organizing pneumonia  Lung opacity | 2 (1.9)  1 (0.9)  1 (0.9)  1 (0.9)  1 (0.9)  1 (0.9)  1 (0.9)  1 (0.9) |
| **Renal and urinary disorders** | **9 (8.4)** |
| Renal impairment  Renal disorder  Hematuria | 5 (4.7)  3 (2.8)  1 (0.9) |
| **General disorders and administration site conditions** | **8 (7.5)** |
| Pyrexia  Face edema  Feeling abnormal  Malaise  Peripheral edema  Mucosal hemorrhage | 4 (3.7)  1 (0.9)  1 (0.9)  1 (0.9)  1 (0.9)  1 (0.9) |
| **Skin and subcutaneous disorders** | **7 (6.5)** |
| Rash  Acute febrile neutropenic dermatosis  Dry skin  Erythema multiforme  Pruritus  Rash maculopapular | 3 (2.8)  1 (0.9)  1 (0.9)  1 (0.9)  1 (0.9)  1 (0.9) |
| **Nervous system disorders** | **5 (4.7)** |
| Cerebral hemorrhage  Altered state of consciousness  Dizziness  Posterior reversible encephalopathy syndrome | 2 (1.9)  1 (0.9)  1 (0.9)  1 (0.9) |
| **Cardiac disorders** | **4 (3.7)** |
| Cardiac failure  Cardiomyopathy  Extrasystoles  Cardiac dysfunction | 2 (1.9)  1 (0.9)  1 (0.9)  1 (0.9) |
| **Musculoskeletal and connective tissue disorders** | **3 (2.8)** |
| Myalgia  Fasciitis | 2 (1.9)  1 (0.9) |
| **Vascular disorders** | **2 (1.9)** |
| Hypertension  Hemorrhage | 1 (0.9)  1 (0.9) |
| **Psychiatric disorders** | **1 (0.9)** |
| Insomnia | 1 (0.9) |
| **Injury, poisoning, and procedural complications** | **1 (0.9)** |
| Subcutaneous hematoma | 1 (0.9) |

ADR, adverse drug reaction; ALT, alanine aminotransferase; AST, aspartate aminotransferase; SOC, system organ class.

**Table S3. Incidence of serious adverse events**

| **Serious AEs, *n* (%)** | ***N*=107** |
| --- | --- |
| Any serious AE | 59 (55.1) |
| **Blood and lymphatic system disorders** | **28 (26.2)** |
| Bone marrow failure  Febrile neutropenia  Neutropenia  Pancytopenia  Thrombocytopenia  Eosinophilia | 13 (12.1)  11 (10.3)  4 (3.7)  3 (2.8)  2 (1.9)  1 (0.9) |
| **Infections and infestations** | **26 (24.3)** |
| Pneumonia  Sepsis  Cellulitis  Infections  Bacteremia  Bronchitis  Bronchopulmonary aspergillosis  Cytomegalovirus infection  Herpes zoster infection  Septic shock  Pneumonia fungal  Infective spondylitis  Mycotic endophthalmitis  Device related infection  Campylobacter colitis  Staphylococcal infection  Adenoviral hemorrhagic cystitis  Cornybacterium bacteremia | 6 (5.6)  5 (4.7)  3 (2.8)  1 (0.9)  1 (0.9)  1 (0.9)  1 (0.9)  1 (0.9)  1 (0.9)  1 (0.9)  1 (0.9)  1 (0.9)  1 (0.9)  1 (0.9)  1 (0.9)  1 (0.9)  1 (0.9)  1 (0.9) |
| **Investigations** | **18 (16.8)** |
| Platelet count decreased  White blood cell count decreased  Neutrophil count decreased  Electrocardiogram QT prolonged  AST increased  ALT abnormal  AST abnormal  Hemoglobin decreased  Aspergillus test positive  C-reactive protein increased | 8 (7.5)  5 (4.7)  4 (3.7)  3 (2.8)  1 (0.9)  1 (0.9)  1 (0.9)  1 (0.9)  1 (0.9)  1 (0.9) |
| **Hepatobiliary disorders** | **7 (6.5)** |
| Liver function abnormal  Liver disorder  Cholangitis | 4 (3.7)  3 (2.8)  1 (0.9) |
| **Gastrointestinal disorders** | **6 (5.6)** |
| Gastrointestinal hemorrhage  Nausea  Upper gastrointestinal hemorrhage  Lower gastrointestinal hemorrhage  Enterocolitis | 2 (1.9)  1 (0.9)  1 (0.9)  1 (0.9)  1 (0.9) |
| **Respiratory, thoracic, and mediastinal disorders** | **6 (5.6)** |
| Interstitial lung disease  Pneumonia aspiration  Pulmonary alveolar hemorrhage  Pulmonary edema  Organizing pneumonia | 2 (1.9)  1 (0.9)  1 (0.9)  1 (0.9)  1 (0.9) |
| **Nervous system disorders** | **5 (4.7)** |
| Cerebral hemorrhage  Altered state of consciousness  Dizziness  Spinal subdural hematoma | 2 (1.9)  1 (0.9)  1 (0.9)  1 (0.9) |
| **Skin and subcutaneous disorders** | **3 (2.8)** |
| Acute febrile neutropenic dermatosis  Erythema multiforme  Rash maculopapular | 1 (0.9)  1 (0.9)  1 (0.9) |
| **Cardiac disorders** | **2 (1.9)** |
| Cardiac failure | 2 (1.9) |
| **Metabolism and nutrition disorders** | **2 (1.9)** |
| Diabetes mellitus  Hypokalemia  Iron overload | 1 (0.9)  1 (0.9)  1 (0.9) |
| **Renal and urinary disorders** | **1 (0.9)** |
| Renal impairment | 1 (0.9) |
| **Musculoskeletal and connective tissue disorders** | **1 (0.9)** |
| Fasciitis | 1 (0.9) |
| **Surgical and medical procedures** | **1 (0.9)** |
| Central venous catheterization | 1 (0.9) |

AE, adverse event; ALT, alanine aminotransferase; AST, aspartate aminotransferase.

**Table S4. Patients who underwent transplantation during the surveillance period**

| **Parameter, *n* (%)** | **Allogeneic HSCT**  **(*N*=14)** |
| --- | --- |
| **Stem cell source** | |
| Peripheral blood  Umbilical cord blood  Bone marrow | 6 (42.9)  4 (28.6)  4 (28.6) |
| **Donor** | |
| Relative  Non-relative | 5 (35.7)  9 (64.3) |
| **HLA match** | |
| Perfect match  Partial match | 6 (42.9)  8 (57.1) |
| **Engraftment** | |
| Yes  No | 13 (92.9)  1 (7.1) |

HLA, human leukocyte antigen; HSCT, hematopoietic stem cell transplantation.
